# Georgia Dental Society

**Published:** 1859-10

**Authors:** 


*Georgia Dental Society.*—We learn from the Macon State Press, that at a preliminary meeting held at Macon, Ga., Friday, July 1st, nearly all of the principal towns in the State being represented, a permanent organization was effected under the above designation.

The officers elected for the ensuing year are, Dr. D. S. Chase, of Augusta, President; Drs. F. Y. Clark, of Savannah, and G. W. Emerson, of Macon, 1st and 2d Vice-Presidents; Dr. W. F. Lee, of Columbus, Recording Secretary; Dr. E. Parsons, of Savannah, Corresponding Secretary, and Dr. J. Fogle, of Columbus, Treasurer.

We are glad that the dentists of Georgia have united their efforts to advance the interests and elevate the dignity of their profession, and we sincerely hope that this may be the result of the organization.

